# Tumor Heterogeneity and Consequences for Bladder Cancer Treatment

**DOI:** 10.3390/cancers13215297

**Published:** 2021-10-22

**Authors:** Etienne Lavallee, John P. Sfakianos, David J. Mulholland

**Affiliations:** 1 Department of Urology, Icahn School of Medicine at Mount Sinai, New York, NY 10029, USA; etienne.lavallee.2@ulaval.ca (E.L.); john.sfakianos@mountsinai.org (J.P.S.); 2 Department of Oncological Sciences, Icahn School of Medicine at Mount Sinai, New York, NY 10029, USA

**Keywords:** bladder cancer, plasticity, intratumoral heterogeneity, treatment resistance, tumoral heterogeneity

## Abstract

**Simple Summary:**

Bladder cancer is a heterogeneous disease that is composed of epithelia with varying transcriptional, mutational and lineage signatures. The epithelia of bladder tumors can also undergo pronounced changes in transcriptional and phenotypical qualities in response to progression, treatment related stresses and cues from the tumor microenvironment (TME). We hypothesize that changes in epithelial tumor heterogeneity (EpTH) occur due to the evolving content of epithelial subpopulations through both Darwinian and Lamarckian-like natural selection processes. We further conjecture that lineage-defined subpopulations can change through nongenomic and genomic cellular mechanisms that include cellular plasticity and acquired driver mutations, respectively. We propose that such processes are dynamic and contribute towards clinical treatment challenges including progression to drug resistance. In this article, we assess mechanisms that may support dynamic tumor heterogeneity with the overall goal of emphasizing the application of these concepts to the clinical setting.

**Abstract:**

Acquired therapeutic resistance remains a major challenge in cancer management and associates with poor oncological outcomes in most solid tumor types. A major contributor is tumor heterogeneity (TH) which can be influenced by the stromal; immune and epithelial tumor compartments. We hypothesize that heterogeneity in tumor epithelial subpopulations—whether de novo or newly acquired—closely regulate the clinical course of bladder cancer. Changes in these subpopulations impact the tumor microenvironment including the extent of immune cell infiltration and response to immunotherapeutics. Mechanisms driving epithelial tumor heterogeneity (EpTH) can be broadly categorized as mutational and non-mutational. Mechanisms regulating lineage plasticity; acquired cellular mutations and changes in lineage-defined subpopulations regulate stress responses to clinical therapies. If tumor heterogeneity is a dynamic process; an increased understanding of how EpTH is regulated is critical in order for clinical therapies to be more sustained and durable. In this review and analysis, we assess the importance and regulatory mechanisms governing EpTH in bladder cancer and the impact on treatment response.

## 1. Introduction

Tumor heterogeneity (TH) has been referred to as the “Rosetta Stone” of cancer progression and therapeutic response [1,2]. The importance of TH for tumor progression and clinical intervention can be demonstrated in preclinical model systems [3,4,5] and in patient tumor samples [6,7,8]. TH can be defined as variation in histological, cellular, and genetic components throughout an individual tumor (intratumoral heterogeneity) or between tumors from different patients (intertumoral heterogeneity).

Within a single tumor, TH encompass both the tumor microenvironment, including stromal and immune cells, as well as the cell autonomous epithelial compartment. TH is also regulated by acellular components such as stromal and connective tissues. Together, such elements form a plastic tumor milieu which can change dynamically during tumor progression and in response to therapeutic challenges.

When considered on a patient population scale, TH is typically extensive and contributes towards the complexity of understanding for when and how often to administer a drug for optimal clinical response. In some instances, drugs that are considered beneficial on a population scale can induce adverse disease progression on certain tumor types, thus highlighting the need for assigning tumor-specific treatments.

While bladder cancer presents as a mutational disease, genetic changes continue to occur in pretreated tumor progression and in response to therapy. Thus, the mutational landscape not only has implications for immune cell infiltration but contributes towards the pool of cells capable of clonal expansion, treatment resistant disease and metastasis. Growing evidence supports that lineage transdifferentiation is an important mechanism for cancer cells to adapt to the stress of therapy [9,10] including acquisition of mesenchymal properties or phenotypes with neuroendocrine qualities. Thus, we propose that oncogenic mutations, genetic diversity, clonal evolution and cellular plasticity are drivers of epithelial heterogeneity in bladder cancer (EpTH) [11].

Recently, single-cell technologies have greatly leveraged our ability to understand the remarkable transcriptional diversity present in bladder cancers. Such technologies provide opportunities to understand the evolutionary changes in tumor cell composition commencing from pretreated primary tumors through to post-treatment, resistant metastasis. We will address the importance of single-cell technologies to increase our understanding of EpTH both with respect to bulk changes in subpopulations as well as single-cell changes in cellular identity (lineage transformation). Improved single-cell techniques will provide the sensitivity and spatial information needed to address critical questions of whether EpTH can be experimentally manipulated to enhance treatment and reduce lethal phenotypes including bladder cancers with neuroendocrine-like (NE-like) signatures. While such processes have proven to be reversible under certain experimental conditions [12,13], the potential to modulate TH in the clinical setting remains to be determined.

As the importance of TH and relevance for tumor progression encompass a large body of information, our discussion and analysis will focus on the tumor epithelial compartment.

## 2. Implications of Patient-Patient Tumor Heterogeneity for Therapeutic Intervention

Clinical bladder cancer typically presents with substantial pathological heterogeneity and a high mutational burden [14]. Multiple transcriptomic classification systems have been proposed to better categorize both muscle invasive bladder cancer (MIBC) and non-muscle-invasive bladder cancer (NMIBC). In MIBC, these systems have included molecular subtyping based on gene signatures that define the cellular lineage qualities including those with basal, luminal, squamous, or neuroendocrine properties [8,15,16,17,18,19,20]. These classification systems were developed based on different datasets of RNA sequencing and gene expression array profiles and used different clustering methodologies. This has resulted in multiple classifiers, the inconsistent use of subtype definitions and limited their use in the stratification of patients for progression or treatment decisions.

In an effort to reconcile previously published MIBC classification schemes, large-scale studies conducted by Kamoun et al. analysed 1750 transcriptomic profiles from 18 datasets and have identified a consensus molecular classification of MIBC that includes six molecular subtypes: luminal papillary (LumP), luminal nonspecified (LumNS), luminal unstable (LumU), stroma-rich, basal/squamous (BaSq) and NE-like [21].

In this instance, consensus classifiers were associated with distinct mRNA signatures which strongly associated with clinical outcome and overall survival [21]. The utility of classifying pre-treatment MIBC molecular subtypes includes the patient selection for cisplatin based neoadjuvant chemotherapy (NAC) [8,22].

Strikingly, only approximately 30% to 40% of residual bladder tumors following NAC or immunotherapy were of the same lineage subtype as the pretreatment tumors [23,24]. In a cohort of 20 chemo resistant matched pre- and post-NAC specimens, resistance to chemotherapy was associated with the development of a p53 pathway signature in the post-treatment specimens [8]. These data underscore that within a pretreated bladder cancer, tumor subpopulations have differential growth kinetics and response to clinical therapies. Moreover, these data highlight the tremendous changes in lineage composition occurring because of therapy in a high percentage of tumors [8]. Finally, previous investigations using warm autopsy specimens revealed tremendous mutational evolution in patient matched primary and metastatic tumor samples [25]. Further understanding of such temporal changes in mutational burden and how these relate to treatment response will help apply appropriate therapies during tumor evolution.

Molecular analysis using bulk RNA seq data has increased our understanding of TH and the potential clinical utility in selecting patients for different systemic treatment [26]. However, a major drawback of this classification strategy is the inability to easily assess individual tumor subpopulations either qualitatively or quantitatively. Data obtained from bulk analysis often produce a predominant lineage signature without the consideration of secondary signatures or tumor subpopulations [27].

The use of single transcriptomic technologies such as single-cell RNA sequencing (scRNA-seq), single-cell DNA seq (scDNA-seq) and spatial sequencing (sp-seq) in research will prove superior for defining clinically viable information, over bulk molecular analysis, by allowing the ability to study TH and to characterize rare cell epithelial subpopulations [28,29]. However, at present, these approaches are both costly and typically require weeks of processing, thus making their application for clinical use challenging.

## 3. Intratumoral Heterogeneity Is Prevalent in Pretreated Primary Bladder Tumors

Intratumoral molecular and genetic heterogeneity have been associated with poor prognosis in multiple cancers including lung cancer, head and neck cancers and chronic lymphocytic leukemia [1,6,7,30,31,32]. In bladder cancer panel of 83 cystectomy specimens’ with significant heterogeneity in molecular subtypes were observed with the basal-squamous subtype being most prevalent [27]. In our recent studies, we have applied single-cell RNA sequencing (scRNA-seq) to analyse transcriptome profiles at the cellular level using a preclinical mouse model of carcinogen (BBN) induced bladder cancer [33]. We showed that tumor epithelia can simultaneously express gene signatures of more than one lineage subtype. Using triple-labeling immunofluorescence, we identified single, double, and triple-lineage marker-positive cells [33]. We made similar observations in our analysis of primary human bladder tumors [33]. Recently, Sirab et al. used dual GATA3/KRT5/6 immunohistochemistry to demonstrate high intratumoral heterogeneity in bladder cancer of the Ba/Sq subtype [34].

Together, these findings demonstrate significant intratumoral heterogeneity in bladder cancer and suggest that categorical molecular subtyping may not be adequate for optimal therapeutic outcomes.

## 4. Mechanisms Regulating Tumor Heterogeneity

Mechanisms promoting Intra-EpTH in pretreated bladder tumors can be broadly divided into mutational and nonmutational. Mutational mechanisms induce heterogeneity in genomic DNA which in turn can translate to phenotypic heterogeneity. Nonmutational mechanisms of heterogeneity induce phenotypic variability without any genetic modifications [35]. The presence of Intra-EpTH and its ability to promote cellular evolution provides a significant limitation or barrier to the long-term durability of targeted therapies. The role of mutational and non-mutational drivers of EpTH in treatment resistance is discussed in the following sections.

## 5. Genetic Heterogeneity in Bladder Tumor Epithelia

Pretreated urothelial carcinoma is characterized by a high mutational burden that contributes to TH. With a median of 8.1 mutations per mega base pair, bladder cancer ranked 11th in terms of mutational burden when compared with 166 other cancer types [14]. Phylogenic analysis, using next-generation targeted sequencing of metachronous bladder tumors showed that urothelial cancer growth follows a branching evolution with a common ancestral origin [36,37]. As new mutations are introduced through different mechanisms, multiple intratumoral subclone populations with varying genomic, phenotypic, and transcriptomic profiles accumulate and contribute towards EpTH [37].

Multiple genetic driver mutations are associated with bladder cancer carcinogenesis and progression [38]. Cells with such mutations are often spatially distributed in a heterogeneous manner [39] between the superficial and deep tissue components of muscle invasive bladder tumors [40]. In FGFR3 mutant cells, sensitivity to FGFR targeted therapies such as erdafitinib may be variable across different epithelia including distinct lineage defined subpopulations. In this instance, treatment mayselect for cell types negative for FGFR3 mutants, thus altering the lineage composition. The potential for these individual epithelial populations to respond and adapt to therapy are areas of clinical importance requiring further investigation using single-cell technologies.

Drivers of bladder cancers with high mutational burden remain poorly understood. Although DNA replication occurs with high fidelity, occasional transcriptional errors still occur even in the absence of mutagens, thereby introducing de novo genetic mutations. This stochastic mutational process is involved in tumorigenesis and disease progression [41]. Exposure to certain carcinogens such as cigarette smoke, radiation or chemicals is also known to induce DNA damage and to play a role in the initiation and the progression of bladder cancer.

The apolipoprotein B mRNA-editing enzyme catalytic polypeptide-like (APOBEC) can induce tumoral genetic heterogeneity leading to an APOBEC mutational signature present in urothelial cancers and other solid tumors [42]. Using whole-exome sequencing of 412 invasive high-grade urothelial tumors, Robertson et al. identified an APOBEC-signature and showed that ABOPEC3 is a major driver of mutagenesis in bladder cancer. Strikingly, they found that more than two thirds of the detected mutations were associated with the APOBEC-signature [15,42]. Moreover, more than half of the APOBEC-signature mutations were clonal and occurred early in the disease process [15]. APOBEC also plays a role in urothelial cancer mutagenesis following chemotherapy [25]. While the clinical importance of APOBEC remains incompletely defined, high levels of APOBEC-mediated mutagenesis in urothelial cancer has been associated with disease progression and higher tumor stage [43,44]. Interestingly, others have shown APOBEC to associate with better clinical outcomes [15,45]. These observations may be partially explained by the varying functions of the seven APOBEC3 family members on tumoral immune response [46]. Another compelling hypothesis is that, beyond a certain level, excess APOBEC-mediated mutagenesis leads to increased antigen presentation and immune activation, thus, resulting in decreased tumor fitness and increased response to therapy [47]. Taken together, this suggests that APOBEC plays a significant role in the genetic evolution of urothelial cancer and that modulation of APOBEC activity could influence disease progression. Indeed, several strategies are available to inhibit APOBEC expression and function including Protein Kinase A inhibition [48] or single-stranded DNA analogues [49].

## 6. Mutational Heterogeneity and Treatment Response

**Figure 1:**
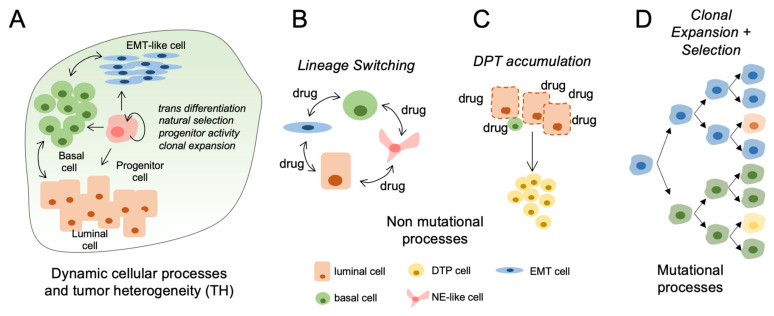
Mechanisms and impact of tumor heterogeneity in clinical bladder cancer progression. (**A**) Pretreated primary bladder tumors are heterogeneous consisting of lineage defined subpopulations with potential to change in relative proportions during progression or treatment. (**B**) Bladder cancer cells can acquire transcriptional and phenotype changes (EMT, NE-like) in response to therapeutic stress. (**C**) Drug tolerant persisters (DTPs) increase in abundance during clinical therapies. This process may be accelerated using drugs at maximum tolerated dosages (MTD). (**D**) Acquired driver mutations can promote the clonal expansion of cell populations having more aggressive progression kinetics and reduced response to therapies.

The genetic heterogeneity in tumor epithelial plays a significant role in disease progression and development of drug resistance largely due to either de novo or acquired therapy-resistant clones [1]. High intratumoral genetic heterogeneity has been associated with poor clinical survival in multiple cancers [6,50]. This was exemplified in an analysis of 77 urothelial tumors from 38 patients, showing that aggressive disease associates with higher mutational heterogeneity [51]. The mechanism for these observations likely relates to cancer treatments exerting a Darwinian-like selection pressure on total cell populations, after which subpopulations most capable of survival persist and ultimately expand to form lethal disease (Figure 1D). In triple negative breast cancer (TNBC), Kim et al. used scRNA-seq and scDNA-seq to explore mechanisms of chemotherapy resistance and showed that resistant genotypes were pre-existing and adaptatively selected by chemotherapy [52]. Whole-exome sequencing in metastatic bladder cancer demonstrates that chemotherapy creates a selection pressure for pre-existing resistance associated gene signatures and is associated with a significant change in the mutational landscape. This analysis showed that only 28% of mutations were common between pre- and postchemotherapy matched samples [25]. Longitudinal DNA sequencing of metastatic breast cancer patients showed that systemic treatment resulted in a genetic bottleneck event and selection of resistant subclones [53]. Together, this data suggests that tumors with high pretreatment TH increase the chances that a pre-existent, resistant subclone will be present and survive the selection pressure induced by treatment, thus resulting in cancer recurrence [54,55].

**Figure 2:**
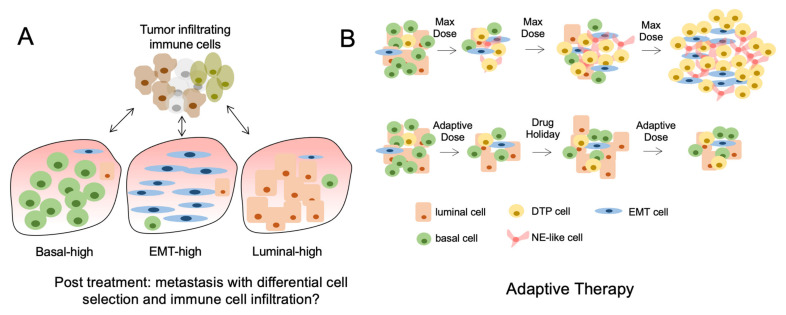
Tumor heterogeneity and the consequences of treatment. (**A**) The lineage composition of post-treatment metastatic tumors can differ significantly because of nonmutational and mutational mechanisms causing differential subpopulation selection. This results in altered immune cell infiltration and response to immune therapies. (**B**) The use of maximum tolerated dose (MTD) of drugs causes initial tumor regression but rapid acquisition of drug resistant tumor cells or DTPs (top). Conversely, the cycled use of drugs at lower doses can prolong the effects of cancer therapies and reduce the acquisition of DTPs (bottom).

Although mutational burden has been associated with response to immune checkpoint inhibitors (ICI), recent evidence suggests that tumors with high Intra-TH have decreased response to ICI. Using a preclinical melanoma mouse model, investigators evaluated the association of total mutational burden and intra-TH with respect to antitumor immunity [56]. They showed that increased intra-TH was associated with decreased antitumor immunity and increased tumor aggressiveness independent of TMB. Clinically, high intra-TH was associated with decreased survival and decreased response to ICI in patients with melanoma [56]. These observations may be explained by findings demonstrating that immune-mediated cell rejection does not occur when tumoral antigens are expressed on a small fraction of tumor cells [57]. This suggest that high intra-TH is associated with neoantigens being expressed by a smaller proportion of cells compared to more homogeneous tumors, thus leading to decreased immune rejection and decreased response to ICI (Figure 2A).

## 7. Nonmutational Mechanisms of Tumor Heterogeneity

As opposed to mutational mechanisms where variability in cellular DNA is the source of heterogeneity, nonmutational drivers of tumoral heterogeneity do not involve modification in the cellular genetic code. Embryological development is an example of nonmutational process of changing heterogeneity where cells with the same DNA code differentiate into multiple cellular phenotypes. Different from genetic heterogeneity, tumor cells contributing toward nonmutational heterogeneity can be highly plastic and have important contributions towards tumor development [1,58].

At a baseline or pretreatment setting, fluctuating cellular transcription constitutes a source of cellular heterogeneity. Upon the exposure to drugs, cells meeting a threshold of resistance-related gene expression will survive and be selected for [59,60]. For example, Shaffer et al. showed that in melanoma, cells undergoing continuous drug exposure can evolve from a state of transcriptional fluctuation to a stable drug-refractory state through epigenetic modification [60]. Interestingly, if drug exposure is stopped before the surviving cells acquire drug-resistance mutations, they can regain their baseline transcriptional variability and revert to a drug-sensitive state. Such changes in transcriptional levels provide a framework to explain retreatment responses observed clinically in different cancers and underscores the impact of nonmutational heterogeneity in cancer treatment resistance [61,62,63]. In prostate cancer, persistent exposure to androgen deprivation leads to a decrease in androgen receptor signaling. However, subsequent re-exposure to testosterone before the development of an androgen-independent state will induce reappearance of androgen receptor function and maintain a castration sensitive state [64]. Clinically, this has resulted in the use of intermittent androgen deprivation therapy (ADT) for the treatment of biochemical recurrence and has been shown to delay progression to a castrate-resistant status while limiting total ADT exposure [65].

## 8. Evidence for Cellular Plasticity and Implications for Treatment Response in Bladder Tumors

Cellular plasticity represents the capacity of cells to adopt different phenotypes and switch between different lineage identities [66]. Together, plasticity mechanisms involve reactivation of developmental cellular programs and include epithelial-mesenchymal transition (EMT), acquisition of cancer stem-cell properties and transdifferentiation potential [12,58,59,67]. Over the last decade, the standard stem-cell model of hierarchical tumor development has been challenged by cell lineage-tracing studies demonstrating that hierarchical cellular plasticity was more common than initially thought and that committed progenitor cells retain the capacity to revert back to a multipotent stem-like phenotype [68,69,70,71,72] (Figure 1A). Cellular plasticity of cancer cells adds further complexity to the concept of tumoral heterogeneity as plasticity is both reversible and can evolve either through dedifferentiation or transdifferentiation [12,13]. In de-differentiation, cells with differentiated properties such as polarity and epithelial marker expression revert back to a less differentiated state within the same cell lineage [73]. During transdifferentiation, a differentiated cell converts to another type of differentiated cell lineage [73,74] (Figure 1B).

In bladder cancer, cellular plasticity has been observed by Yang et al. using single-cell sequencing to demonstrate that nonstem cells, in urothelial cancers, can acquire stem-like properties and develop self-renewal capabilities [75]. Cellular plasticity and phenotype switching has also been observed in patient-derived bladder cancer organoids [76]. Our previous studies have used the in vitro culture of tumor cells isolated from a carcinogen (-BBN) induced mouse model of bladder cancer, to show that cell populations enriched for Epcam and CD49f expression have enhanced capacity to undergo microenvironment-independent plasticity. We also demonstrated cell lineage plasticity of human muscle invasive bladder cancer in an in vivo mouse model [33]. An important body of literature also supports the presence of cellular plasticity in breast cancer. Flow cytometry isolation was used to isolate three mammary epithelial cell states and demonstrate that luminal and basal cells can revert back to a functional stem-like phenotype and regenerate all three cell phenotype linage using both in vitro and in vivo models [77]. Further studies support the presence of cellular plasticity in other solid tumors such as colon cancer, breast cancer and lung cancer [78,79,80,81,82]. In general, cellular plasticity is thought to be dynamically involved at the invasive edge of tumor cells as they undergo cytoskeletal changes in order to accommodate cellular expansion and invasion [83].

## 9. Epithelial-Mesenchymal Transition (EMT)

EMT is a tightly regulated process involved in embryonal development and in tissue healing and represents the most studied example of cellular plasticity [84]. EMT should not be regarded as a strictly binary process as cells are usually in a continuum of transitional states between epithelial and mesenchymal phenotype [85]. Cancer cells with mesenchymal properties are characterised by loss of polarity, decreased cell-to-cell adhesion and increase in migration capabilities [86,87]. In bladder cancer, tumoral RNA expression of EMT markers such as N-cadherin, Vimentin, Slug and Snail have been linked to increased clinical stage, grade, and worse clinical outcome [88,89]. Weak expression of E-cadherin and strong expression of MMP-9 and TWIST, measured by immunohistochemistry, were also shown to be independently associated to tumor recurrence in a cohort of 161 non-muscle-invasive bladder cancer [90]. Mesenchymal characteristics of circulating tumor cells have been linked to cancer progression and resistance to treatment in breast cancer patients [91]. In human bladder cancer cell lines, the transcription factor TWIST, a known marker of EMT, has been associated with anthracycline resistance [92].

## 10. Neuroendocrine-Like Phenotypes

The formation of epithelia with neuroendocrine qualities constitutes another lineage occurring frequently during therapeutic stress [93] (Figure 1B). For example, in prostate cancer, prolonged exposure to androgen pathway inhibitors induce the development of aggressive disease characterized by low androgen receptor expression and response to most standards of care treatments [93,94]. In bladder cancer, transcriptomes from 34 small cell bladder cancer were compared to 84 conventional urothelial cancer specimens to study the role of epithelial transition in bladder cancer. Analysis of mRNA and miRNA transcriptome profiles suggested that bladder cancer progression to the more aggressive small cell variant was driven by dysregulated EMT leading to an epithelial to neuronal lineage plasticity [95]. Mechanisms underlying development of small-cell bladder cancer are incompletely understood, but it has been suggested that anticancer treatment such as chemotherapy could induce transdifferentiation to a neuroendocrine phenotype, similar to what has been observe in prostate cancer and hormone therapy [26]. These data suggest that multiple types of therapies have the potential to induce sufficient cellular stress to cause changes in lineage identity.

Taken together, this suggests that plasticity is important in the evolution of bladder cancer. We conjuncture that plasticity represents a clinically important resistance mechanism as cells switch from a treatment-sensitive lineage to treatment-resistant lineages in order to accommodate the stress of therapy.

## 11. Reversal of Cellular Plasticity as a Therapeutic Strategy

While mounting evidence supports that lineage plasticity is a bona fide mechanism for cancer cells to adapt to therapy, less is understood as to whether plasticity can be reversed in a controlled manner. Due to its important role in tumor progression and aggressiveness, EMT represents an attractive target for the development of targeted therapies. Multiple pathways regulating EMT are being investigated as potential treatment targets with clinical trials investigating drugs capable of targeting EMT being conducted in solid tumors such as breast cancer, lung cancer and colorectal cancer [96,97]. Interestingly, purposed for other clinical purposes, such as the analgesic etodolac and the cholesterol lowering agent, simvastatin, possess some inhibitory effects on EMT and are currently evaluated for roles in cancer treatment [98]. While incompletely understood, in vitro studies on bladder cancer cells suggest that simvastatin may alter EMT by deactivating the PI3K/AKT and the MAPK/ERK signalling pathways [99]. We suggest that the use of EMT targeting agents will lead to increased durability of current clinical therapies when used in combination [99]. Despite these opportunities, challenges in targeting cellular transition states include identifying which patients to treat and when, during clinical progression. Currently strategies have considered assessing bloodborne CTCs or even secreted markers expressed in the sera.

## 12. Drug-Tolerant Persisters and Post-Treatment Lineage Heterogeneity

In response to drug exposure, a subpopulation of cancer cells termed drug-tolerant persisters (DTPs) will acquire a poorly differentiated phenotype and enter a dormant, slow-cycling and drug tolerant state [59] (Figure 1C). DTPs can survive in a quiescent state for prolonged periods of time. A similar phenomenon is observed in bacteria, where a subpopulation of cells exhibits reversible antibiotic-tolerant properties and can survive treatment [100].

Although tolerant cells may pre-exist in the tumor, it has been shown that some cells become DTPs through EMT and phenotype plasticity following drug exposure [101,102,103].While there is currently no consensus on specific markers associated with DTPs [103], the accumulation of DTPs can occur in different tumor types by seemingly different mechanisms.

In basal-like breast cancer cell lines, exposure to MEK and PI3K/mTOR inhibitor resulted in development of DTPs through epithelial plasticity driven by therapeutic challenge [104]. In EGFR mutant non-small-cell lung cancer (NSCLC) cell lines treated with an EGFR TKI, DTPs represented 0.3–5% of cell population. Once drug exposure was stopped, DTPs resumed growth and reacquired EGFR TKI sensitivity, suggesting a nonmutational mechanism of resistance [105]. DTPs can survive for months, allowing time for the development of new resistant clones through the acquisition of driver mutations and creating a link between nonmutational and mutational resistance mechanisms [103,106]. Therefore, an increased understanding of cellular plasticity and of the mechanisms surrounding the development DTPs will be key in preventing cancer drug tolerance.

## 13. Follow Up

Tumor heterogeneity is a dynamic process that serves an important role in tumor progression and response to therapies. Changes in TH through clinical evolution are likely a consequence of both changing proportions of lineage defined subpopulations as well as processes of cellular plasticity. Understanding how clinically approved therapies regulate these processes on a temporal scale will help define therapies. Adaptive therapy is an example of a novel treatment approach that incorporates these concepts and aims to maintain a population of treatment-sensitive cells to prevent the expansion of treatment-resistant cell populations (Figure 2B).

Using mouse models of breast cancer, Enriquez-Navas et al. showed that low doses of paclitaxel are effective in controlling tumor volume and preventing unopposed proliferation of treatment-resistant cell populations [107]. Adaptive therapy is also being explored with hormonal therapy in delaying castration-resistant prostate cancer [108]. Directed plasticity is another interesting approach where the aim of treatment is to guide cellular plasticity to induce transdifferentiation of malignant cells to a benign phenotype [109,110]. In bladder cancer, development of therapies focussing on cellular plasticity mechanisms could force cellular lineage switching from malignant phenotypes to more benign and indolent phenotypes. Therapies preventing plasticity towards a DPT phenotype could also increase long-term response to current treatments by preventing treatment resistance. Preclinical research of this nature is studying the transcriptional repressor of CDK7/12, THZ1, in mouse models of bladder cancer [111].

A better understanding of cancer cell heterogeneity and plasticity will allow clinicians to consider tumor heterogeneity in a dynamic and adaptive fashion, rather than in a static manner in the elaboration of new treatment approach.

## 14. Conclusions

Tumor heterogeneity and cellular plasticity are hypothesized to play important roles in the evolution and management of bladder cancer. The limitations of homogenous and static classifications of urothelial cancer need to be acknowledged. The heterogeneous and dynamic nature of this disease should be emphasized paving the way for further research on specific mechanisms regulating the dynamics of tumor subpopulations and cellular plasticity.
